# Supplementary material for: Snapshots of ABCG1 and ABCG5/G8: A Sterol’s Journey to Cross the Cellular Membranes
Source: Int J Mol Sci. 2022 Dec 28;24(1):484. doi: 10.3390/ijms24010484 (PMC9820320; doi:10.3390/ijms24010484)
Supplement: Supplementary file 1 [file ijms-24-00484-s001.zip › ijms-2036762-supplementary.pdf]

Human ABCG1 Sequence

```
>sp|P45844|ABCG1_HUMAN ATP-binding cassette sub-family G member 1 OS=Homo
sapiens OX=9606 GN=ABCG1 PE=1 SV=3 (NP_004906.3)
MACLMAAFSVGTAMNASSYSAEMTEPKSVCVSVDEVVSSNMEATETDLLNGHLKKVDNNL
TEAQRFSSLPRRAAVNIEFRDLSYSVPEGPWWRKKGYKTLLKGISGKFNSGELVAIMGPS
GAGKSTLMNILAGYRETGMKGAVLINGLPRDLRCFRKVSCYIMQDDMLLPHLTVQEAMMV
SAHLKLQEKDEGRREMVKEILTALGLLSCANTRTGSLSGGQRKRLAIALELVNNPPVMFF
DEPTSGLDSASCFQVVSLMKGLAQGGRSIICTIHQPSAKLFELFDQLYVLSQGQCVYRGK
VCNLVPYLRDLGLNCPTYHNPADFVMEVASGEYGDQNSRLVRAVREGMCDSDHKRDLGGD
AEVNPFLWHRPSEEVKQTKRLKGLRKDSSSMEGCHSFSASCLTQFCILFKRTFLSIMRDS
VLTHLRITSHIGIGLLIGLLYLGIGNEAKKVLSNSGFLFFSMLFLMFAALMPTVLTFPLE
MGVFLREHLNYWYSLKAYYLAKTMADVPFQIMFPVAYCSIVYWMTSQPSDAVRFVLFAAL
GTMTSLVAQSLGLLIGAASTSLQVATFVGPVTAIPVLLFSGFFVSFDTIPTYLQWMSYIS
YVRYGFEGVILSIYGLDREDLHCDIDETCHFQKSEAILRELDVENAKLYLDFIVLGIFFI
SLRLIAYFVLRYKIRAER
```

Sequence Alignment (5 representative mammalian species with human sequence in bold):
→ Conserved polar residues are highlighted based on conservation scores obtained from analysis and alignment of 950 mammalian ABCG1 sequences using **BLASTp.** The conservation scores were obtained by Consurf (https://consurf.tau.ac.il/consurf_index.php).

Highlighted residues conservation score key:

- conservation score of 7
- conservation score of 8
- conservation score of 9

***The residues chosen as the involved residues in the ABCG1 Polar relay that displayed polar linkages in a 3D setting.**

```
XP_030661937.1      MACLMAAFSVGTAMNASSYSAEMTEPKSVCVSVDEVVSSNMEAAETDLLNGHLKKVDNNL      60
XP_004062897.1      MACLMAAFSVGTAMNASSYSAEMMEPKSVCVSVDEVVSSNMEATETDLLNGHLKKVDNNL      60
PNJ42434.1          MACLMAAFSVGTAMNASSYSAEMMEPKSVCVSVDEVVSSNMEATETDLLNGHLKKVDNNL      60
NP_004906.3         MACLMAAFSVGTAMNASSYSAEMTEPKSVCVSVDEVVSSNMEATETDLLNGHLKKVDNNL      60
XP_003319138.2      MACLMAAFSVGTAMNASSYSAEMTEPKSVCVSVDEVVSSNMEATETDLLNGHLKKVDNNL      60

XP_030661937.1      TEAQRFSSLPRRAAVNIEFRDLSYSVPEGPWWRKKGYKTLLKGISGKFSSGELVAIMGPS      120
XP_004062897.1      TEAQRFSSLPRRAAVNIEFRDLSYSVPEGPWWRKKGYKTLLKGISGKFNSGELVAIMGPS      120
PNJ42434.1          TEAQRFSSLPRRAAVNIEFRDLSYSVPEGPWWRKKGYKTLLKGISGKFNSGELVAIMGPS      120
NP_004906.3         TEAQRFSSLPRRAAVNIEFRDLSYSVPEGPWWRKKGYKTLLKGISGKFNSGELVAIMGPS      120
XP_003319138.2      TEAQRFSSLPRRAAVNIEFRDLSYSVPEGPWWRKKGYKTLLKGISGKFNSGELVAIMGPS      120

XP_030661937.1      GAGKSTLMNILAGYRETGMKGAVLINGLPRDLRCFRKVSCYIMQDDMLLPHLTVQEAMMV      180
XP_004062897.1      GAGKSTLMNILAGYRETGMKGAVLINGLPRDLRCFRKVSCYIMQDDMLLPHLTVQEAMMV      180
PNJ42434.1          GAGKSTLMNILAGYRETGMKGAVLINGLPRDLRCFRKVSCYIMQDDMLLPHLTVQEAMMV      180
NP_004906.3         GAGKSTLMNILAGYRETGMKGAVLINGLPRDLRCFRKVSCYIMQDDMLLPHLTVQEAMMV      180
XP_003319138.2      GAGKSTLMNILAGYRETGMKGAVLINGLPRDLRCFRKVSCYIMQDDMLLPHLTVQEAMMV      180

XP_030661937.1      SAHLKLQEKDEGRREMVKEILTALGLLSCANTRTGSLSGGQRKRLAIALELVNNPPVMFF      240
XP_004062897.1      SAHLKLQEKDEGRREMVKEILTALGLLSCANTRTGSLSGGQRKRLAIALELVNNPPVMFF      240
PNJ42434.1          SAHLKLQEKDEGRREMVKEILTALGLLSCANTRTGSLSGGQRKRLAIALELVNNPPVMFF      240
NP_004906.3         SAHLKLQEKDEGRREMVKEILTALGLLSCANTRTGSLSGGQRKRLAIALELVNNPPVMFF      240
XP_003319138.2      SAHLKLQEKDEGRREMVKEILTALGLLSCANTRTGSLSGGQRKRLAIALELVNNPPVMFF      240
```

```
XP_030661937.1          DEPTSGLDSASCFQVVSLMKGLAQGGRSIICTIHQPSAKLFELFDQLYVLSQGQCVYRGK      300
XP_004062897.1          DEPTSGLDSASCFQVVSLMKGLAQGGRSIICTIHQPSAKLFELFDQLYVLSQGQCVYRGK      300
PNJ42434.1              DEPTSGLDSASCFQVVSLMKGLAQGGRSIICTIHQPSAKLFELFDQLYVLSQGQCVYRGK      300
NP_004906.3             DEPTSGLDSASCFQVVSLMKGLAQGGRSIICTIHQPSAKLFELFDQLYVLSQGQCVYRGK      300
XP_003319138.2          DEPTSGLDSASCFQVVSLMKGLAQGGRSIICTIHQPSAKLFELFDKLYVLSQGQCVYRGK      300

XP_030661937.1          VCNLVPYLRDLGLNCPTYHNPADFVMEVASGEYGDQNSRLVRAVREGMCDSDHKRDLGGD      360
XP_004062897.1          VCNLVPYLRDLGLNCPTYHNPADFVMEVASGEYGDQNSRLVRAVREGMCDSDHKRDLGGD      360
PNJ42434.1              VCNLVPYLRDLGLNCPTYHNPADFVMEVASGEYGDQNSRLVRAVREGMCDSDHKRDLGGD      360
NP_004906.3             VCNLVPYLRDLGLNCPTYHNPADFVMEVASGEYGDQNSRLVRAVREGMCDSDHKRDLGGD      360
XP_003319138.2          VCNLVPYLRDLGLNCPTYHNPADFVMEVASGEYGDQNSRLVRAVREGMCDSDHKRDLGGD      360

XP_030661937.1          AEVNPFLWHRPSEEVKQTKRLKGLRKDSSSMEGCHSFSASCLTQFCILFKRTFLSIMRDS      420
XP_004062897.1          AEVNPFLWHRPSEEVKQTKRLKGLRKDSSSVEGCHSFSASCLTQFCILFKRTFLSIMRDS      420
PNJ42434.1              AEVNPFLWHRPSEEVKQTKRLKGLRKDSSSMEGCHSFSASCLTQFCILFKRTFLSIMRDS      420
NP_004906.3             AEVNPFLWHRPSEEVKQTKRLKGLRKDSSSMEGCHSFSASCLTQFCILFKRTFLSIMRDS      420
XP_003319138.2          AEVNPFLWHRPSEEVKQTKRLKGLRKDSSSMEGCHSFSASCLTQFCILFKRTFLSIMRDS      420

                             *  *                                              *
XP_030661937.1          VLTHLRITSHIGIGLLIGLLYLGIGNEAKKVLSNSGFLFFSMLFLMFAALMPTVLTFPLE      480
XP_004062897.1          VLTHLRITSHIGIGLLIGLLYLGIGNEAKKVLSNSGFLFFSMLFLMFAALMPTVLTFPLE      480
PNJ42434.1              VLTHLRITSHIGIGLLIGLLYLGIGNEAKKVLSNSGFLFFSMLFLMFAALMPTVLTFPLE      480
NP_004906.3             VLTHLRITSHIGIGLLIGLLYLGIGNEAKKVLSNSGFLFFSMLFLMFAALMPTVLTFPLE      480
XP_003319138.2          VLTHLRITSHIGIGLLIGLLYLGIGNEAKKVLSNSGFLFFSMLFLMFAALMPTVLTFPLE      480

                                             **  *   *
XP_030661937.1          MGVFLREHLNYWYSLKAYYLAKTMADVPFQIMFPVAYCSIVYWMTSQPSDAVRFVLFAAL      540
XP_004062897.1          MGVFLREHLNYWYSLKAYYLAKTMADVPFQIMFPVAYCSIVYWMTSQPSDAVRFVLFAAL      540
PNJ42434.1              MGVFLREHLNYWYSLKAYYLAKTMADVPFQIMFPVAYCSIVYWMTSQPSDAVRFVLFAAL      540
NP_004906.3             MGVFLREHLNYWYSLKAYYLAKTMADVPFQIMFPVAYCSIVYWMTSQPSDAVRFVLFAAL      540
XP_003319138.2          MGVFLREHLNYWYSLKAYYLAKTMADVPFQIMFPVAYCSIVYWMTSQPSDAVRFVLFAAL      540

                            *   **
XP_030661937.1          GTMTSLVAQSLGLLIGAASTSLQVATFVGPVTAIPVLLFSGFFVSFDTIPTYLQWMSYIS      600
XP_004062897.1          GTMTSLVAQSLGLLIGAASTSLQVATFVGPVTAIPVLLFSGFFVSFDTIPTYLQWMSYIS      600
PNJ42434.1              GTMTSLVAQSLGLLIGAASTSLQVATFVGPVTAIPVLLFSGFFVSFDTIPTYLQWMSYIS      600
NP_004906.3             GTMTSLVAQSLGLLIGAASTSLQVATFVGPVTAIPVLLFSGFFVSFDTIPTYLQWMSYIS      600
XP_003319138.2          GTMTSLVAQSLGLLIGAASTSLQVATFVGPVTAIPVLLFSGFFVSFDTIPTYLQWMSYIS      600

XP_030661937.1          YVRYGFEGVILSIYGLDREDLHCDIDETCHFQKSEAILRELDVENAKLYLDFIVLGIFFI      660
XP_004062897.1          YVRYGFEGVILSIYGLDREDLHCDIDETCHFQKSEAILRELDVENAKLYLDFIVLGIFFI      660
PNJ42434.1              YVRYGFEGVILSIYGLDREDLHCDIDETCHFQKSEAILRELDVENAKLYLDFIVLGIFFI      660
NP_004906.3             YVRYGFEGVILSIYGLDREDLHCDIDETCHFQKSEAILRELDVENAKLYLDFIVLGIFFI      660
XP_003319138.2          YVRYGFEGVILSIYGLDREDLHCDIDETCHFQKSEAILRELDVENAKLYLDFIVLGIFFI      660

                          *
XP_030661937.1          SLRLIAYFVLRYKIRAER 678
XP_004062897.1          SLRLIAYFVLRYKIRAER 678
PNJ42434.1              SLRLIAYFVLRYKIRAER 678
NP_004906.3             SLRLIAYFVLRYKIRAER 678
XP_003319138.2          SLRLIAYFVLRYKIRAER 678
```
